# Parkinson's Disease in Pregnancy: A Case Report and Review of the Literature

**DOI:** 10.3389/fneur.2019.01349

**Published:** 2020-02-19

**Authors:** Sara Olivola, Serena Xodo, Enrica Olivola, Fabiana Cecchini, Ambrogio Pietro Londero, Lorenza Driul

**Affiliations:** ^1^Department of Gynaecology and Obstetrics, School of Medicine of Udine, Udine, Italy; ^2^IRCCS Istituto Neurologico Mediterraneo (INM) Neuromed, Pozzilli, Italy

**Keywords:** Parkinson's disease, pregnancy, L-Dopa, literature review, case report

## Abstract

**Background:** Pregnancy in Parkinson's disease is a rare occurrence, and to date, clinical experience with its management is rather limited. In clinical practice, doubts concern mainly the impact of PD on gestation, labor, and delivery as well as the safety of dopaminergic drugs.

**Case and review of the literature:** We report the case of a 40-year-old woman with an 8-year history of PD. In the first trimester of her pregnancy, her motor status was similar to the pre-conceptional period. In gestation week 16, her motor status dramatically worsened and she complained of predictable “off” periods in the afternoon. For this reason, her dose of L-DOPA/carbidopa was increased up to 500/125 mg per day. At 39 gestational weeks, she gave birth to a healthy girl with an Apgar score of 9 by an uncomplicated cesarean delivery. The child was not breast fed to avoid exposure to antiparkinsonian drugs. The L-DOPA/carbidopa dosage remained constant during the postpartum period. We performed a systematic review of the literature using Ovid Medline, Scopus, and PubMed (including Cochrane database). We used the search terms “Parkinson disease” AND “pregnancy.” We identified 20 studies of PD in pregnancy with a total of 37 pregnant women with PD. The most important available data concern the safety of L-DOPA therapy during pregnancy. There seems to be some risk of worsening of the condition or upcoming of new PD symptoms during or shortly after pregnancy.

**Conclusion:** More data concerning the safety of antiparkinsonian drugs in PD treatment, as well as the effect of pregnancy on parkinsonian symptoms are needed. According to the current state of the art, L-DOPA therapy should be considered preferable to other drugs during pregnancy.

## PRÉCIS

Parkinson disease in pregnancy and its management

## Teaching Points

1. Parkinson disease is a rare occurrence during pregnancy.
2. The change in PD symptoms during pregnancy is unpredictable and there is poor knowledge about the correct management of pregnancy in PD.
3. According to the current literature, L-DOPA therapy should be the best option for patients in terms of safety and efficacy.

## Introduction

Parkinson's disease (PD) is the second most common neurodegenerative disorder. It is caused by the degeneration of dopaminergic neurons in the substantia nigra pars compacta of the midbrain (1). While PD is usually thought as a disease characterized by cardinal motor symptoms including resting tremor, slowness motor (bradykinesia), and rigidity, a wide range of non-motor symptoms such as autonomic, sensory, sleep, and neuropsychiatric dysfunctions are now widely recognized as part of the clinical manifestations (2). PD frequently affects elderly people (>60 years), and its prevalence is heavily age-dependent, but ~5% of patients diagnosed with PD are under 40 years of age (3–5).

PD in young people is frequently under-diagnosed, but the interest in the disease onset among younger people is constantly growing. In addition, there is a somewhat greater incidence of PD in males than females, with an approximate ratio of 1.5:1 reported in literature (6–8). The reason for this gender difference is still unclear, but it supports the hypothesis that estrogen exerts a neuroprotective action through antioxidative, anti-inflammation, and anti-apoptotic pathways (7, 9, 10). Estrogen also enhances dopaminergic functions by increasing dopamine synthesis and its release, by inhibiting dopamine re-uptake and by modulating dopamine receptors density and sensitivity, as demonstrated in animal studies (11, 12). Nevertheless, the exact role of estrogens in dopaminergic functions needs to be further clarified (13, 14).

Considering these epidemiological features, pregnancy in PD is a rare occurrence, and to date, clinical experience with its management is rather limited. In clinical practice, doubts concern mainly the impact of PD on gestation, labor, and delivery, as well as the safety of dopaminergic drugs. Moreover, the change in PD symptoms during pregnancy is unpredictable and there is still very poor knowledge among obstetricians about the correct management of pregnancy in PD (15). Unfortunately, the available evidence from reported cases is mainly formed by retrospective questionnaires or analyses of patients with PD who have been pregnant (16–19). In any case, the majority of cases reported in the literature ended in successful full-term deliveries.

## Case

We report the case of a 40-year-old woman with an 8-year history of PD. The disease began with resting tremor and rigidity of her left upper extremity. There was no history of tobacco, drug, or alcohol consumption. She became pregnant for the first time when she was on antiparkinsonian therapy. She referred to us when she was 39 years old. A brain magnetic resonance performed at the beginning of her motor symptoms was normal. Genetic testing to investigate the monogenic form of PD was not performed because of the patient's refusal. She responded well to treatment as assessed by the Unifield Parkinson's Disease Rating Scale (UPDRS) motor section (part III), the most widely used measure to assess motor symptoms and signs in PD (20). She had a UPDRS part III score of 39 in the “off” status (phases with important motor symptoms and no response to medication) assessed in the morning before therapy assumption and a score of 22 in the “on” state (stages with good response to medication and few symptoms) after medication intake. When she became pregnant, she was taking 300/75 mg/day of L-DOPA/carbidopa and her motor examination revealed asymmetrical bilateral tremor, rigidity, and bradykinesia predominant on the left side. In the first trimester of her pregnancy, her motor status was similar to the pre-conceptional period. In gestation week 16, her motor status dramatically worsened (stronger rigidity, resting tremor, and onset of gait disturbance such as shuffling with her left leg) and she complained of predictable “off” periods in the afternoon. For this reason, the dose of L-DOPA/carbidopa was increased up to 500/125 mg per day. At 39 gestational weeks, she gave birth to a healthy girl with an Apgar score of 9 by an uncomplicated cesarean delivery chosen instead of a vaginal delivery because of her motor impairment. General and neurological examination of the baby revealed no abnormalities and the routine blood tests were normal. The child was not breast fed to avoid exposure to antiparkinsonian drugs, but medical information on this topic is lacking. The L-DOPA/carbidopa dosage remained constant during the postpartum period and the patient's neurological status is currently stable.

## Systematic Review

We performed a systematic review of the literature using Ovid Medline, Scopus, and PubMed (including Cochrane database). Relevant articles describing any case of PD in pregnancy were identified from the above databases without any time, language, or study limitations. We used the search terms “Parkinson disease” AND “pregnancy.” All non-duplicate identified articles were independently reviewed by two authors (S.O. and S.X.), and relevant articles were selected by mutual agreement. Studies were deemed eligible for inclusion in our review if they described at least one case of PD in pregnancy. We analyzed the age of patients, medication, change in PD symptoms during pregnancy, perinatal outcome, and breastfeeding.

We identified 20 studies of PD in pregnancy with a total of 38 pregnant women with PD and 53 pregnancies (Supplementary Data 1–3). Therapy in PD is symptomatic and includes L-DOPA usually combined with a peripheral dopa-decarboxilase inhibitor (carbidopa or benserazide), monoamine oxidase-B (MAO-B) inhibitors (selegine and rasagiline), dopamine agonists commonly subdivided into ergot derivatives (bromocriptina, pergolide, cabergolina, and lisuride), and non-ergot derivatives (pramipexole, ropinirole, rotigotine, and apomorphine), amantadine, catechol-O-methyltransferase inhibitors (COMT) (entacapone or polecapone), and anticholinergic drugs. All antiparkinsonian medications are contraindicated during pregnancy and are regarded by the Food and Drug Administration (FDA) as Pregnancy Category C, due to the lack of human or animal evidence regarding their consequences on fetal development (21).

### L-DOPA and Dopa-Decarboxylase Inhibitors (Table 1)

There is no doubt that levodopa is the most efficient medication for PD. Many studies use L-DOPA alone or combined with dopa-decarboxylase inhibitors (carbidopa or benserazide) for PD in pregnancy. Of the 38 women with PD in pregnancy, only 14 use L-DOPA in monotherapy or combined with dopa-decarboxylase inhibitors (36.8%). Although some animal studies (28, 29) have shown that high doses of L-DOPA in monotherapy or combined with peripheral dopa-decarboxylase inhibitors may lead to intrauterine growth retardation or teratogenic effects as malformation of the circulatory system and skeleton malformation (15), several reports hypothesized that the use of L-DOPA in humans during pregnancy might be safe (16–18, 22–25). There is only one case report in the literature on the use of continuous intestinal infusion of L-DOPA/carbidopa gel treatment during pregnancy, after a percutaneous gastrojejunostomy (27). L-DOPA crosses the placenta and is found in fetal tissues (30). By contrast, carbidopa and benserazide are found at high concentrations in the umbilical cord, but in very low concentrations in fetal tissue, indicating that the placenta works as a defense barrier (28–30). Animal studies have shown that L-DOPA treatment was not associated with a deleterious effect on pregnancy and on fetus. However, two cases of miscarriage in the first trimester (23, 26) and one case of fetal osteomalacia were reported during L-DOPA in monotherapy or with dopa-decarboxylase treatment (15).

**Table 1:** L-DOPA for PD in pregnancy in monotherapy or combined with decarboxylase inhibitors.

	**References**	**Medication during pregnancy**	**Change of therapy**
1a	Cook and Klawans (22)	L-DOPA/CARBIDOPA	Increase L-DOPA dosage during pregnancy
2b		L-DOPA	None
2c		L-DOPA	None
3a	Gershanik and Leist (23)	L-DOPA/CARBIDOPA	None
3b		L-DOPA/CARBIDOPA	None
4	Golbe (16)	L-DOPA/CARBIDOPA	None
5		L-DOPA/CARBIDOPA	None
6		L-DOPA/CARBIDOPA	None
7		L-DOPA/CARBIDOPA	None
8a		L-DOPA/CARBIDOPA	None
8b		L-DOPA/CARBIDOPA	None
9	Allain et al. (17)	L-DOPA/BENSERAZIDE	None
10	Ball and Sagar (18)	L-DOPA/CARBIDOPA	None
11	Shulman et al. (24)	L-DOPA/CARBIDOPA	Increase L-DOPA dosage during pregnancy
12a	Campos-Sousa et al. (25)	L-DOPA	None
12b		L-DOPA	None
12c		L-DOPA	None
12d		L-DOPA	None
13	Robottom et al. (26)	L-DOPA	None
14	Zlotnik et al. (27)	L-DOPA/CARBIDOPA INTESTINAL GEL (LCIG)	None

### Dopamine Agonists (Table 2)

Few successful reports, nine studies in literature (15, 16, 31–37), indicate that dopamine agonists (such as bromocriptine, pergolide, pramipexole, cabergolina, and ropinirole) have been used in pregnancy in monotherapy or in association with L-DOPA alone or with dopa-decarboxylase inhibitors without adverse effects on fetal development (31–34, 36, 37). Studies conducted on mice have shown that Pergolide is associated with a 7% reduction of average fetal body weight of the mother, but there is no evidence of any harm to their fetuses (38). Bromocriptine has been widely used to facilitate pregnancy in women with hyperprolactinemia (39). No evidence of teratogenic effect or major complications of gestation were reported with the use of bromocriptine in human or animals (31, 39). There is no report of a teratogenic effect of pramipexole therapy during pregnancy in animals and there is no data with the use of rotigotine or apomorphine in pregnancy.

**Table 2:** Use of dopamine agonist for PD in pregnancy.

	**References**	**Medication during pregnancy**	**Change of therapy during pregnancy**
1	Golbe (16)	BROMOCRIPTINE	None
2	Hagell et al. (15)	L-DOPA/BENSERAZIDE + BROMOCRIPTINE	Bromocriptine was discontinued in the 2nd month of pregnancy During pregnancy was increased L-DOPA dosage
3	Benito-Leon et al. (31)	BROMOCRIPTINE	None
4	De Mari et al. (32)	L-DOPA + PERGOLIDE	None
5a	Scott and Chowedhury (33)	L-DOPA/CARBIDOPA + CABERGOLINE	None
5b		L-DOPA/CARBIDOPA + CABERGOLINE	None
6	Mucchiut et al. (34)	PRAMIPEXOLE	None
7	Lindh (35)	L-DOPA/CARBIDOPA + ENTACAPONE + BROMOCRIPTINA	None
8	Asha et al. (36)	L-DOPA/CARBIDOPA, BROMOCRIPTINA + ROPINIROLE	None
9	Bernbir et al. (37)	PRAMIPEXOLE	None

### MAO-B Inhibitors (Table 3)

MAO-B inhibitors (selegine and rasagiline) are used as monotherapy or as added therapy. Animal studies of MAO-B inhibitors have evidenced mild to seriously harmful neurobehavioral effects (19). No study has been published on treatment with selegine alone in pregnant animals. Only two studies (19, 40) report the use of selegine associated with L-DOPA in woman with PD in pregnancy, with no evidence of harmful effect for the fetus and the mother.

**Table 3:** Use of MAO-B inhibitors for PD in pregnancy.

	**References**	**Medications during pregnancy**	**Change of therapy during pregnancy**
1	Kupsch and Oertel (19)	L-DOPA/BENSERAZIDE + SELEGINE	None
2	Serikawa et al. (40)	L-DOPA/CARBIDOPA until 19th week, then ENATCAPONE+ SELEGINE	Increase dose of L-DOPA/carbidopa

### COMT Inhibitors (Table 4)

COMT is an enzyme for peripheral metabolism of levodopa. COMT inhibitors are always used in association with levodopa and carbidopa. There is only one report in the literature that uses COMT inhibitor in association with selegine starting at the 19th week of pregnancy in a spontaneous dichorionic/diamniotic twin pregnancy, with the delivery of healthy male twins (40).

**Table 4:** Use of COMT inhibitors for PD in pregnancy.

	**Reference**	**Medications during pregnancy**	**Change of therapy during pregnancy**
1	Serikawa et al. (40)	L-DOPA/CARBIDOPA + ENTACAPONE + SELEGINE	L-DOPA/CARBIDOPA until 19th week, then + ENATCAPONE + SELEGINE

### Amantidine (Table 5)

Amantidine is originally introduced as an antiviral medication. It was accidentally found that the drug is able to relieve PD early symptoms. Amantidine seems to be teratogenic in both animals and humans (42), because it increases the risk of complications and malformations during pregnancy (especially in the first trimester). Amantidine should be avoided before conception and during gestation and breast feeding. Skeletal developmental defects have been reported in the offspring of rats receiving amantidine in high doses (42). We found two studies (16, 41) using amantidine in monotherapy or combined with other medications in pregnant women with PD.

**Table 5:** Use of amantidine for PD in pregnancy.

	**References**	**Medications during pregnancy**	**Change of therapy during pregnancy**
1	Nora et al. (41)	AMANTIDINE	None
2	Golbe (16)	L-DOPA/CARBIDOPA + AMANTIDINE	None
		L-DOPA + AMANTIDINE	None
		AMANTIDINE	None
		AMANTIDINE + TRIHEXYPHENIDYL	None

### Anticholinergics (Table 6)

Clinical use of anticholinergics is limited due to adverse effects. In the few reports on anticholinergic drugs in pregnant women with PD (16), they are used either as monotherapy or as adjunctive treatment. Trihexyphenidyl, diphenhydramine, procyclidine, benztropine mesylate, hydrochloride, and biperiden hydrochloride or antihistaminic derivatives such as diparalene are commonly used. Most anticholinergics are pregnancy category B2, but their safety in pregnancy has not been well-established (21). Except for one case of spontaneous miscarriage and a case of probable molar pregnancy, all pregnancies were successful (16).

**Table 6:** Use of anticholinergics for PD in pregnancy.

	**References**	**Medications during pregnancy**	**Change of therapy during pregnancy**
1	Golbe (16)	TRIHEXYPHENIDYL	None
		PROCICLIDINE	None
		TRIHEXYPHENIDYL + DIPHENHYDRAMINE	None
		TRIHEXYPHENIDYL + DIPHENHYDRAMINE	None
		TRIHEXYPHENIDYL + DIPHENHYDRAMINE	None
		AMANTIDINE + TRIHEXYPHENIDYL	None

In the limited literature available, the effect of pregnancy on PD symptoms seems to be quite controversial (Table 7). In 9 of the 53 pregnancies described (17.0%), no antiparkinsonian drug was used (16, 22, 26, 27). Two studies (two pregnant women) did not evaluate the change of PD symptoms during pregnancy (23, 41). In 28 (52.8%) out of 53 pregnancies, symptoms did not change during pregnancy (16, 18, 22, 25–27, 31–33, 35–37); in 3 women (5.7%), some improvement was evidenced (16, 19); and in 21 cases (39.6%), a significant worsening of PD during pregnancy was described (15–17, 23, 24, 26, 27, 34, 40). About complications of pregnancy in women suffering from PD and treated with antiparkinsonian agents during gestation, Table 7 summarizes the results found in the literature. All studies reported pregnancy complications except one study (41). Most women (68.6%) completed their pregnancy without complications (15–19, 22–27, 31–37). Described complications were Trisomy 21 in one case (16), placenta abruption in one case (33), preeclampsia and dystocia in one case (16), and depression with nausea and vomiting in one case (16). The most common complication was miscarriage (8.0%) (16, 23, 26) and spotting in the first trimester (6.0%) (16, 22).

**Table 7:** Change in PD symptoms and complications during pregnancy.

	**References**	**Medications during pregnancy**	**Change in PD during pregnancy**	**Complications during pregnancy**
1	Nora et al. (41)	AMANTIDINE	NS	NS
2a	Cook and Klawans (22)	None	None	None
2b		L-DOPA/CARBIDOPA	None	Spotting in the 1st trimester
3a		L-DOPA	None	None
3b		L-DOPA	None	None
4a	Gershanik and Leist (23)	L-DOPA/CARBIDOPA	Worsening of “wearing off” effect	None
4b		L-DOPA/CARBIDOPA	NS	Miscarriage at the 2nd month
5	Golbe (16)	L-DOPA/CARBIDOPA	Transient increase in “OFF” time	Spotting in the 1st trimester
6		L-DOPA/CARBIDOPA	Transient worsening of Corhea	None
7		L-DOPA/CARBIDOPA	None	None
8		L-DOPA/CARBIDOPA	Gait worsened	Depression (until delivery), nausea and vomiting at 8–9 months
9a		L-DOPA/CARBIDOPA	Rigidity worsened	None
9b		L-DOPA/CARBIDOPA	None	None (elective abortion at the 1st trimester)
9c		L-DOPA/CARBIDOPA + AMANTIDINE	“ON” state worsened	Preeclampsia
10a		L-DOPA + AMANTIDINE	None	Miscarriage at 4 months
10b		TRIHEXYPHENIDYL	None	None
10c		PROCICLYDINE	None	None
11		None	Tremor began	Dystocia
12		AMANTIDINE	Dexterity improved transiently	Spotting in the 1st trimester
13		DIPARALENE	Rigidity worsened slightly	None
14a		TRIHEXYPHENIDYL + DIPHENHYDRAMINE	Gait slowed	None
14b		TRIHEXYPHENIDYL + DIPHENHYDRAMINE	General rigidity worsened	None
15		None	Bradykinesia began, transient resting tremor during labor	None
16		None	Micrographia, gait, gait difficult began soon after delivery	None
17		None	Dexterity worsened, nausea all pregnancy, depression	None
18		None	None	None
19		TRIHEXYPHENIDYL + DIPHENHYDRAMINE	None	Spontaneous miscarriage in 4 months
20		AMANTIDINE + TRIHEXYPHENIDYL	None	Probable molar pregnancy
21a		None	None	None (elective abortion at 10 weeks)
21b		BROMOCRIPTINE	None	None (elective abortion at 10 weeks)
22		AMITRIPTYLINE	Mood improved	Trisomy 21 on amniocentesis (elective abortion)
23	Allain et al. (17)	L-DOPA/BENSERAZIDE	Motor status worsened, depression began	None
24	Ball and Sagar (18)	L-DOPA/CARBIDOPA	None	None
25	Hagell et al. (15)	L-DOPA/BENSERAZIDE+ BROMOCRIPTINE (bromocriptine discontinued in the 2nd month of pregnancy)	Transient depression, increased L-DOPA dosage	None
26	Kupsch and Oertel (19)	L-DOPA/BENSERAZIDE + SELEGINE	Improving symptoms	None
27	Benito-Leon et al. (31)	BROMOCRIPTINE	None	None
28	Shulman et al. (24)	L-DOPA/CARBIDOPA	Increased motor dysfunctions, increased L-DOPA dosage	None
29	De Mari et al. (32)	L-DOPA + PERGOLIDE	None	None
30	Mucchiut et al. (34)	PRAMIPEXOLE	Motor disability worsened	None
31a	Scott and Chowedhury (33)	L-DOPA/CARBIDOPA + CABERGOLINE	None	None
31b		L-DOPA/CARBIDOPA + CABERGOLINE	None	Placental abruption at 32 weeks
32	Lindh (35)	L-DOPA/CARBIDOPA + ENTACAPONE + BROMOCRIPTINA	None	None
33a	Campos-Sousa et al. (25)	L-DOPA	None	None
33b		L-DOPA	None	None
33c		L-DOPA	None	None
33d		L-DOPA	None	None
34a	Robottom et al. (26)	L-DOPA	None	Spontaneous miscarriage at 11 weeks
34b		None	Motor disability worsened	None
35	Asha et al. (36)	L-DOPA/CARBIDOPA, BROMOCRIPTINE and ROPINIROLE	None	None (IVF pregnancy)
36	Serikawa et al. (40)	L-DOPA/CARBIDOPA until 19th weeks then + ENATCAPONE + SELEGINE	Motor disability worsened	Preterm premature rupture of the membranes
37	Bernbir et al. (37)	PRAMIPEXOLE	None	None
38a	Zlotnik et al. (27)	None	None	None
38b		Levodopa–carbidopaintestinal gel (LCIG)	Severe dyskinesia during labor	None

Analyzing the data in the literature, quite a few case reports showed worsening during intrapartum or postpartum period (16, 24, 26, 27, 34, 40). The main complication in the postpartum period was depression (16, 24) (Table 8). Most of the reported cases showed no teratogenic effect of drugs on the fetus resulting in an uncomplicated birth of a healthy baby. In one case, the use of amantidine was associated with fetal cardiovascular malformation (a single ventricle with pulmonary atresia) (41). carbidopa/levodopa administration was associated with one case of osteomalacia (15). A single case of seizure was reported in a newborn exposed to bromocriptine plus a combination of levodopa, carbidopa, and entacapone (35) and a woman treated with L-DOPA/carbidopa until 19 weeks of pregnancy and then with enatcapone and selegine gave birth to an infant with ventricular septum defect (40). Breast feeding is inhibited by dopamine agonists, because they inhibit physiologic lactation (43). Amantidine is known to enter breast milk in low quantities, but there is no report of adverse effects in breast-fed infants (43, 44). It is not known whether L-DOPA, selegine, or peripheral dopa decarboxylase inhibitors are excreted into breast milk (45, 46).

**Table 8:** Perinatal outcome and breast feeding.

	**References**	**Mode of delivery**	**Complication intrapartum**	**Postpartum period**	**Perinatal outcome**	**Breast feeding**
1	Nora et al. (41)	NS	NS	NS	Single ventricle with pulmonary atresia	NS
2a	Cook and Klawans (22)	Term spontaneous VD	None	NS		None
2b		Term spontaneous VD	None	NS		None
3a		Term spontaneous VD	None	NS		None
3b		Term spontaneous VD	None	NS		None
4	Gershanik and Leist (23)	Term spontaneous VD	None	None		NS
5	Golbe (16)	Term CD	Inadequate progression in labor	None	Healthy	None
6		Term spontaneous VD	None	None	Healthy	None
7		Term spontaneous VD	None	None	Healthy	None
8		Term spontaneous VD	None	None	Healthy	None
9a		Term spontaneous VD	Rigidity worsened	None	Healthy	None
9b		Term spontaneous VD	Preeclampsia	/	Inguinal hernia	None
10a		Term spontaneous VD	None	Mild postpartum depression	Healthy	None
10b		Term spontaneous VD	None	Mild postpartum depression	Healthy	None
11		CD for dynamic dystocia	Dystocia	Post-partum depression for 2–3 days	Healthy	None
12		Term spontaneous VD	None	None	Healthy	None
13		Term spontaneous VD	None	None	Healthy	None
14		Term spontaneous VD	None	None	Healthy	None
15a		Term spontaneous VD	None	Post-partum depression	Healthy	None
15b		Term spontaneous VD	Transient resting tremor during labor	None	Healthy	None
16		Term spontaneous VD	None	Gait difficulty began soon after delivery	Healthy	None
17		Term spontaneous VD	None	None	Healthy	None
18		Forceps delivery	None	None	Healthy	None
19	Allain et al. (17)	Term spontaneous VD	None	None	Healthy^*^	None
20	Ball and Sagar (18)	Forceps delivery + epidural anesthesia	None	None	Healthy	None
21	Hagell et al. (15)	Term elective CD	None	None	Osteomalacia	None
22	Kupsch and Oertel (19)	Term elective CD	None	None	Healthy	None
23	Benito-Leon et al. (31)	Term spontaneous VD	None	None	Healthy	None
24	Shulman et al. (24)	Term spontaneous VD	None	Postpartum depression	Healthy	None
25	De Mari et al. (32)	Term CD (podalic presentation)	None	None	Healthy	None
26	Mucchiut et al. (34)	CD because motor impairment	Motor disability worsened	None	Healthy	None
27a	Scott and Chowedhury (33)	Term spontaneous VD	None	None	Healthy	None
27b		Urgent CD for placental abruption (32 weeks)	None	None	Healthy	None
28	Lindh (35)	Term spontaneous VD	None	None	Generalized seizures^**^	None
29a	Campos-Sousa et al. (25)	Term spontaneous VD	None	None	Healthy	None
29b		Term spontaneous VD	None	None	Healthy	None
29c		Term spontaneous VD	None	None	Healthy	None
29d		Term spontaneous VD	None	None	Healthy	None
30	Robottom et al. (26)	Term spontaneous delivery	Motor disability worsened	None	Healthy	For 6 months
31	Asha et al. (36)	(IVF pregnancy) Term CD for non-reassuring CTG	None	None	Healthy	None
32	Serikawa et al. (40)	CD at 35 weeks (premature rupture of membranes) D/D twin pregnancy	Motor disability worsened	None	Ventricular septum defect in one infant	None
33	Bernbir et al. (37)	CD at 36 weeks	None	None	Healthy	None
34a	Zlotnik et al. (27)	Term spontaneous VD (42 weeks)	None	None	Healthy	Yes
34b		Term spontaneous VD	Severe dyskinesia during labor	None	Healthy^***^	For 3 months

* *The newborn was immediately transferred to the newborn intensive care unit and observed for 8 days without any indication of neurological, vegetative, or metabolic abnormality*.

** *One hour after delivery, the infant developed generalized seizures. Phenobarbital was given without effect. Diazepam was added and no further seizures were observed. The baby was also short of breath and had increased CRP, and the chest X-ray showed pneumonia*.

*** *The newborn at 10 months, 0.1st weight percentiles for age (low weight percentile for age during the first year of life)*.

## Comment

PD is an age-related disease, and pregnancy in women with PD is very uncommon. The incidence of PD increases with age, although about 5% is diagnosed before the age of 40 (3). The literature available on pregnancy in PD includes 38 case reports, but the amount of the evidence on the effect of pregnancy on PD and on safety of dopaminergic medications is so far inconclusive. Studies on the impact of pregnancy on PD described no change (16, 18, 22, 25–27, 31–33, 35–37), improvement (16, 19), or worsening (15–17, 23, 24, 26, 27, 34, 40) of the motor symptoms. Non-motor symptoms seem to worsen during pregnancy as well (16). Our patient experienced worsening of her motor symptoms during gestation and a need for higher L-DOPA dosage throughout pregnancy. The rapid deterioration during gestation and the clinical stability in the postpartum period support the idea of a deleterious effect of pregnancy on PD, which is probably determined by hormonal modifications or L-DOPA pharmacokinetic alteration induced by pregnancy or both. The main concern for managing pregnancy in PD is the safety of antiparkinsonian medications for the fetus. The remaining antiparkinsonian medications belongs to category C of FD A (21), which means that animal studies point to some risk, while human studies are either not available or they have not confirmed that risk (39, 42). Based on current knowledge, dopamine agonists do not seem to have abortive or teratogenic effects on pregnancy (25, 31–36). The most important available data concern the safety of L-DOPA therapy during pregnancy. Animal data indicate that L-DOPA with or without dopa-decarboxylase inhibitors (carbidopa or benserazide) induces some teratogenic risk when given in high doses in animal models (39). However, in seven cases where L-DOPA was used as monotherapy, there were no reports of a teratogenic effect (22, 25). However, there were two miscarriages in PD patients using L-DOPA (23, 26) and one case of fetal osteomalacia when L-DOPA was associated with benserazide (15). Amantadine is the only antiparkinsonian medication where administration was associated with cardiovascular malformations in one infant (41). There is no report of major malformations with the use of selegine (19), but data are insufficient. Up to now, there is no data available on rotigotine and apomorphine. As for the risk of using antiparkinsonian drugs during breast feeding, here too evidence is fairly limited. Almost all drugs are excreted into breast milk and are bioavailable to the infant (43), but, generally speaking, concentrations in breast milk are close to or even lower than concentrations in the mother's plasma, and the total dose to which the infant is exposed is usually much lower than the therapeutic dose range.

## Conclusions

It is necessary that obstetricians and neurologists learn how to manage pregnancy in PD patients, to ensure the best possible maternal and fetal outcome. There seems to be some risk of worsening of the condition or upcoming of new PD symptoms during or shortly after pregnancy. Patients with PD considering pregnancy should be informed about this. More data concerning the safety of antiparkinsonian drugs in PD treatment as well as the effect of pregnancy on parkinsonian symptoms are needed. There is also a need for clinical trials to evaluate the use of antiparkinsonian medication in pregnancy. According to the current state of the art, L-DOPA therapy should be considered preferable to other drugs during pregnancy.

## Ethics Statement

Written informed consent was obtained from the patient for publication of this case report as per our standard institutional rules. A copy of the written consent is available from the corresponding author.

## Author Contributions

SO and SX conceived the idea and wrote the manuscript. Together with FC performed the revision of the current literature. These authors mainly gave to the draft an obstetrical perspective. EO wrote the manuscript. She gave an insight into the neurological point of view of this challenging issue. AL performed the statistical calculation. LD corrected and supervised the work. Each author approved the final version of the draft.

### Conflict of Interest

The authors declare that the research was conducted in the absence of any commercial or financial relationships that could be construed as a potential conflict of interest.
