# Supplementary figures and images for: Parkinson's Disease in Pregnancy: A Case Report and Review of the Literature
Source: Front Neurol. 2020 Feb 19;10:1349. doi: 10.3389/fneur.2019.01349 (PMC7042376; doi:10.3389/fneur.2019.01349)

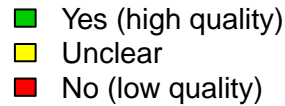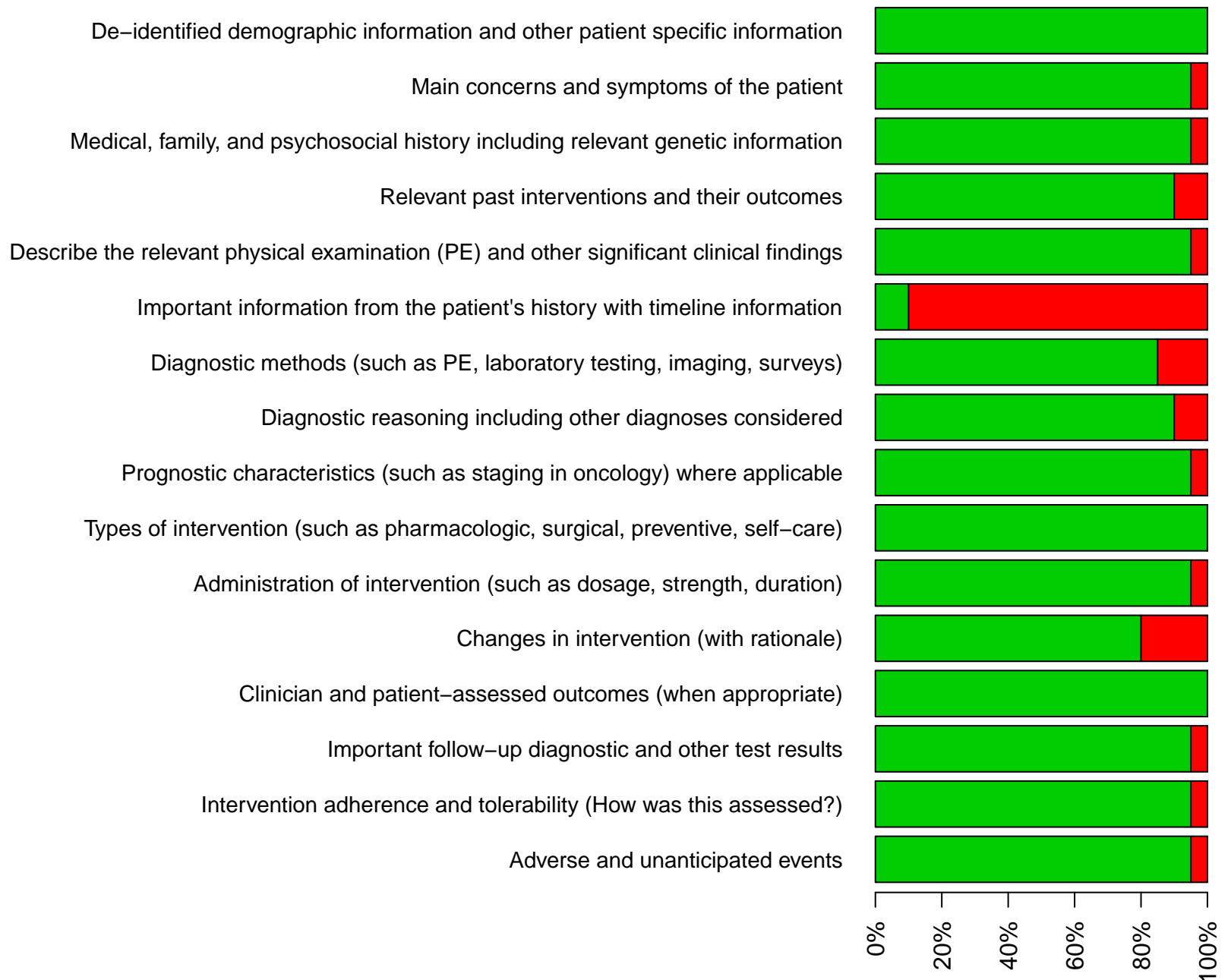

Supplement: Supplementary file 1 [file Data_Sheet_1.PDF]
