# Supplementary material for: Parkinson's Disease in Pregnancy: A Case Report and Review of the Literature
Source: Front Neurol. 2020 Feb 19;10:1349. doi: 10.3389/fneur.2019.01349 (PMC7042376; doi:10.3389/fneur.2019.01349)
Supplement: Supplementary file 2 [file Data_Sheet_2.PDF]

Study	De−identified demographic information and other patient specific information	Main concerns and symptoms of the patient	Medical, family, and psychosocial history including relevant genetic information	Relevant past interventions and their outcomes	Describe the relevant physical examination (PE) and other significant clinical findings	Important information from the patient's history with timeline information	Diagnostic methods (such as PE, laboratory testing, imaging, surveys)	Diagnostic reasoning including other diagnoses considered	Prognostic characteristics (such as staging in oncology) where applicable	Types of intervention (such as pharmacologic, surgical, preventive, self−care)	Administration of intervention (such as dosage, strength, duration)	Changes in intervention (with rationale)	Clinician and patient−assessed outcomes (when appropriate)	Important follow−up diagnostic and other test results	Intervention adherence and tolerability (How was this assessed?)	Adverse and unanticipated events
Zlotnik 2014	+	+	+	+	+	−	+	+	+	+	+	+	+	+	+	+
Bernbir 2014	+	+	+	+	+	−	+	+	+	+	+	+	+	+	+	+
Serikawa 2011	+	+	+	+	+	−	+	+	+	+	+	+	+	+	+	+
Asha 2010	+	+	+	+	+	−	+	+	+	+	+	+	+	+	+	+
Robottom 2008	+	+	+	+	+	+	+	+	+	+	−	+	+	+	−	−
Campos Sousa 2008	+	+	+	+	+	−	+	+	+	+	+	−	+	+	+	+
Lindh 2007	+	+	+	+	+	−	+	+	+	+	+	+	+	+	+	+
Scott 2005	+	+	+	+	+	−	+	+	+	+	+	+	+	+	+	+
Mucchiut 2004	+	+	+	+	+	−	+	+	+	+	+	+	+	+	+	+
De Mari 2002	+	+	+	+	+	−	+	+	+	+	+	+	+	+	+	+
Shulman 2000	+	+	+	+	+	+	+	+	+	+	+	+	+	+	+	+
Benito Leon 1999	+	+	+	+	+	−	+	+	+	+	+	+	+	+	+	+
Kupsch 1998	+	+	+	+	+	−	+	+	+	+	+	+	+	+	+	+
Hagell 1998	+	+	+	+	+	−	+	+	+	+	+	+	+	+	+	+
Ball 1995	+	+	+	+	+	−	−	−	+	+	+	+	+	+	+	+
Allain 1989	+	+	+	+	+	−	+	+	+	+	+	+	+	+	+	+
Golbe 1987	+	−	−	−	+	−	+	−	−	+	+	−	+	+	+	+
Gershanik 1986	+	+	+	+	+	−	+	+	+	+	+	−	+	+	+	+
Cook 1985	+	+	+	+	+	−	−	+	+	+	+	+	+	+	+	+
Nora 1975	+	+	+	−	−	−	−	+	+	+	+	−	+	−	+	+
