# Supplementary material for: Parkinson's Disease in Pregnancy: A Case Report and Review of the Literature
Source: Front Neurol. 2020 Feb 19;10:1349. doi: 10.3389/fneur.2019.01349 (PMC7042376; doi:10.3389/fneur.2019.01349)
Supplement: Supplementary file 3 [file Data_Sheet_3.PDF]

PRISMA 2009 Flow Diagram

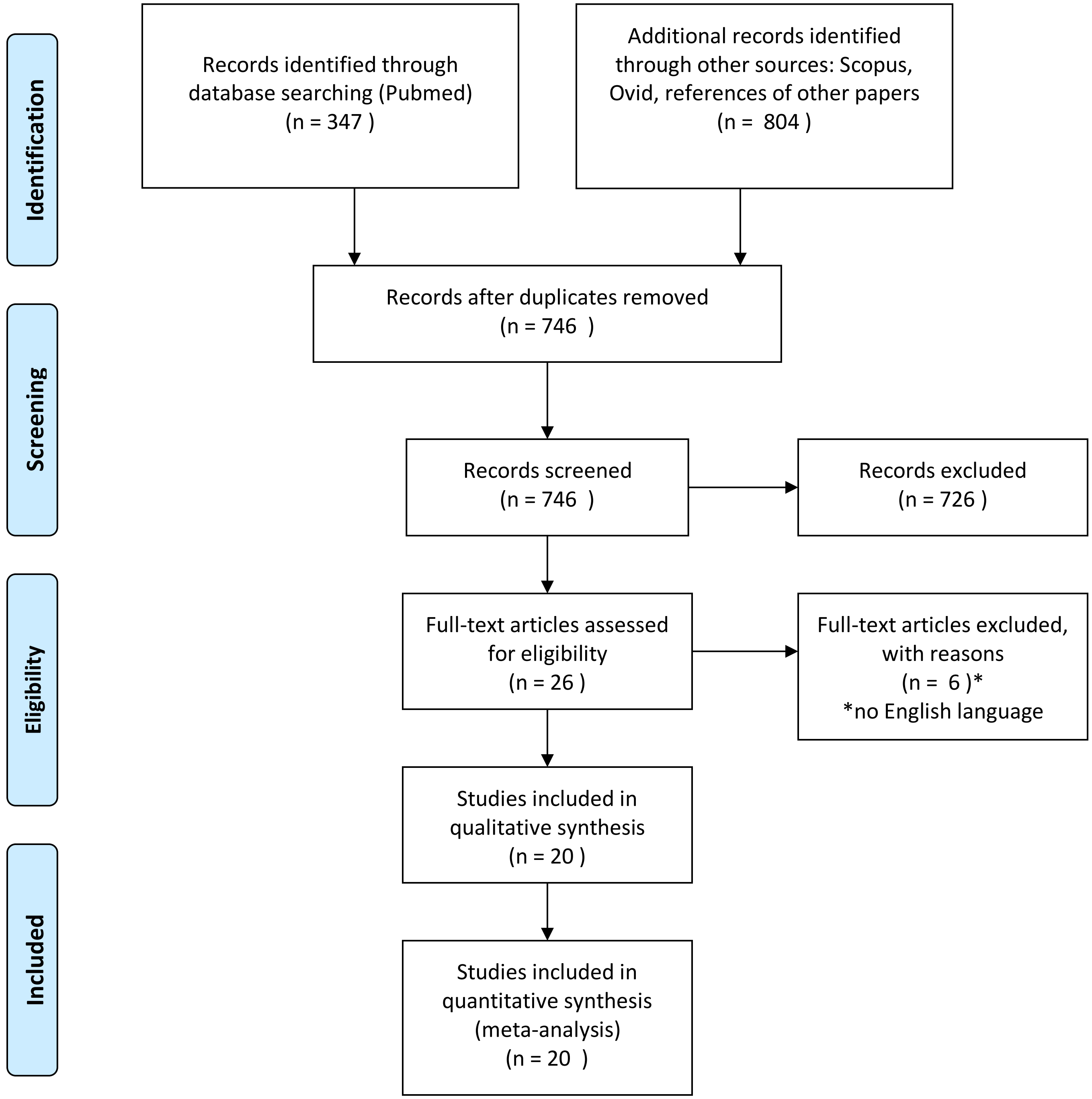

From: Moher D, Liberati A, Tetzlaff J, Altman DG, The PRISMA Group (2009). *P*referred *R*eporting *I*tems for *S*ystematic Reviews and *M*eta-*A*nalyses: The PRISMA Statement. PLoS Med 6(6): e1000097. doi:10.1371/journal.pmed1000097

For more information, visit www.prisma-statement.org.
